# Supplementary material for: MetaRibo-Seq measures translation in microbiomes
Source: Nat Commun. 2020 Jun 29;11:3268. doi: 10.1038/s41467-020-17081-z (PMC7324362; doi:10.1038/s41467-020-17081-z)
Supplement: Supplementary file 10 — Supplementary Data 7 [file 41467_2020_17081_MOESM10_ESM.zip › File2/Confidence_VeryHigh_Taxonomy/15743_out.krona.html]

Javascript must be enabled to view this page.

members
magnitude
magnitudeUnassigned
count
unassigned
taxon
rank

15743\_out

7

superkingdom
7
2

phylum
7
1239

7
class
186801

186802
7
order

31979
family
1

genus
1
1485

1
species
1263069

SRS142781\_contig\_number\_contig-100\_30792.30793

4
family
186803

1432051
1
genus

species
1

SRS146888\_contig\_number\_10222
1432052

1506577
genus
1

1
species
29361

SRS075398\_contig\_number\_contig-100\_1931.22692

1
species
1855376

SRS023715\_contig\_number\_716

572511
genus
1

species
1

SRS057717\_contig\_number\_7384
33038

2
family
541000

2
genus
1263

2
species
1897020

SRS016954\_contig\_number\_contig-100\_234.156402SRS024625\_contig\_number\_20481
